# Molecular Characterization of Microbiota in Cerebrospinal Fluid From Patients With CSF Shunt Infections Using Whole Genome Amplification Followed by Shotgun Sequencing

**DOI:** 10.3389/fcimb.2021.699506

**Published:** 2021-08-20

**Authors:** Paul Hodor, Christopher E. Pope, Kathryn B. Whitlock, Lucas R. Hoffman, David L. Limbrick, Patrick J. McDonald, Jason S. Hauptman, Jeffrey G. Ojemann, Tamara D. Simon

**Affiliations:** ^1^Seattle Children’s Hospital, Seattle, WA, United States; ^2^Department of Pediatrics, University of Washington, Seattle, WA, United States; ^3^New Harmony Statistical Consulting LLC, Clinton, WA, United States; ^4^Department of Neurosurgery, Washington University in St. Louis, St. Louis, MO, United States; ^5^Division of Neurosurgery, University of British Columbia, Vancouver, BC, Canada; ^6^Children’s Hospital Los Angeles, Los Angeles, CA, United States; ^7^Department of Pediatrics, Keck School of Medicine at the University of Southern California, Los Angeles, CA, United States

**Keywords:** cerebrospinal fluid, CSF shunt infection, microbiota, *Staphylococcus epidermidis* CLIMB1, high throughput DNA sequencing

## Abstract

Understanding the etiology of cerebrospinal fluid (CSF) shunt infections and reinfections requires detailed characterization of associated microorganisms. Traditionally, identification of bacteria present in the CSF has relied on culture methods, but recent studies have used high throughput sequencing of 16S rRNA genes. Here we evaluated the method of shotgun DNA sequencing for its potential to provide additional genomic information. CSF samples were collected from 3 patients near the beginning and end of each of 2 infection episodes. Extracted total DNA was sequenced by: (1) whole genome amplification followed by shotgun sequencing (WGA) and (2) high-throughput sequencing of the 16S rRNA V4 region (16S). Taxonomic assignments of sequences from WGA and 16S were compared with one another and with conventional microbiological cultures. While classification of bacteria was consistent among the 3 approaches, WGA provided additional insights into sample microbiological composition, such as showing relative abundances of microbial versus human DNA, identifying samples of questionable quality, and detecting significant viral load in some samples. One sample yielded sufficient non-human reads to allow assembly of a high-quality *Staphylococcus epidermidis* genome, denoted CLIMB1, which we characterized in terms of its MLST profile, gene complement (including putative antimicrobial resistance genes), and similarity to other annotated *S. epidermidis* genomes. Our results demonstrate that WGA directly applied to CSF is a valuable tool for the identification and genomic characterization of dominant microorganisms in CSF shunt infections, which can facilitate molecular approaches for the development of better diagnostic and treatment methods.

## Introduction

Hydrocephalus is a common cause of neurological disability in children (Williams et al., 2007). Cerebrospinal fluid (CSF) shunt placement allows children with hydrocephalus to survive and avoid ongoing brain injury. However, CSF shunts cause new chronic surgical problems, including the need for surgical revisions from catheter obstruction and infections (Kestle, 2003; Simon et al., 2009; Simon et al., 2012). CSF shunt infection treatment usually requires surgical removal of the CSF shunt, two weeks of intravenous antibiotics tailored to organisms recovered from conventional culture, and a second surgery to place a new CSF shunt (Kestle et al., 2006; Simon et al., 2010). Despite this aggressive treatment, reinfection rates range from 20 to 25% (Kulkarni et al., 2001; Kestle et al., 2006; Tuan et al., 2011). Reinfection rates are higher still for children with their second CSF shunt infection (Tuan et al., 2011). An improved understanding of the mechanisms of infection is critical to effectively treat more than 2,000 CSF shunt infections diagnosed each year (Simon et al., 2008).

Among the 20 to 25% of patients with treated CSF shunt infection who develop reinfection, it is unclear whether reinfections are caused by an organism that persists from one infection to the next, or are independent infection events. In the majority of reinfections (70%), the organism(s) recovered by culture differ between the first and second infection (Tuan et al., 2011). Detailed genomic characterization of microorganisms from CSF can help answer questions about the microbial determinants of infection and reinfection.

Several methods exist for characterizing microorganisms in CSF and other clinical specimens. Microbiological culture methods, typically bacterial cultures, are standard in the routine clinical laboratory practice (Baron et al., 2013; Pittman et al., 2014). While sensitive, culture methods are limited to detecting specific human pathogens and often do not provide strain information without additional investigation. Multilocus sequence typing (MLST) can identify strain subtypes for certain species, based on characteristic gene sequence markers (Belén et al., 2009). 16S rRNA sequencing can identify a broader range of species than either culture or MLST, including some not detectable by culture, but is generally less sensitive and often cannot discriminate microbes to the species level (Church et al., 2020). Next-generation sequencing applications are increasingly being used to address the limitations of these traditional methods (Mitchell and Simner, 2019), potentially combined with mass spectrometry (Hrabak et al., 2020). Whole genome sequencing offers advantages such as detailed genomic characterization, strain discrimination, identification of putative antibiotic resistance genes, and characterization of metabolic capacity, all of which are relevant in studying the dynamics of infection and treatment response. However, in the case of CSF shunt infection, samples have low microbial loads, and therefore a DNA enrichment method must be applied prior to sequencing. Whole genome amplification and shotgun sequencing (WGA) is one such method, which was recently applied to a cohort of hospitalized patients with infectious meningitis and encephalitis (Wilson et al., 2019), identifying potential pathogens that were previously underappreciated.

Here we describe a proof of concept study to assess the usefulness of WGA for characterizing the microbiota of CSF shunt infections, comparing results of WGA with those of conventional microbiological culture and 16S rRNA high throughput sequencing (16S).

## Materials and Methods

### Study Subjects

The cohort considered in this study was previously described (Whitlock et al., 2021). Eligible subjects were children ≤18 years old undergoing treatment for conventional culture-confirmed CSF shunt infection at either Seattle Children’s Hospital (SCH) or Primary Children’s Hospital (PCH). Enrollment occurred from 2010 to present at SCH and from 2008 to 2015 at PCH. In this study we considered a subset of children who failed treatment for CSF shunt infection (i.e. had CSF shunt reinfection) and had CSF collected both near the beginning and end of both infection episodes (n = 3).

### CSF Specimen Collection

Collection and storage of CSF was done under standard sterile conditions. The initial CSF sample for diagnosis of infection was obtained by needle aspiration of the shunt reservoir outside the operating room in a bedside “shunt tap”. The CSF sample near the beginning of the infection, which was analyzed in this study, was either left over from the initial diagnostic sample or was obtained in the operating room under sterile conditions from the system being removed during the first surgery to treat infection. Samples near the end of the infection were generally obtained under sterile bedside conditions through a sampling port within sterile extension tubing attached to the external ventricular drain.

CSF samples were stored at 4°C upon collection, aliquoted into vials of ~100 µl, and stored at -70°C. PCH samples were shipped overnight to Seattle on dry ice for analysis.

### Conventional Culture Identification of Bacteria

All samples were tested by routine CSF aerobic culture in hospital-certified laboratories at both SCH and PCH. The methodology followed guidelines of the Clinical and Laboratory Standards Institute guidelines (https://clsi.org).

### DNA Extraction, 16S rRNA Gene Amplification and Sequencing

A diagram of the experimental procedures, beginning with DNA extraction and ending with taxonomic assignment of sequences, is shown in **Supplementary Figure 1**.

DNA was extracted and purified from CSF samples using the AGOWA mag Mini DNA isolation kit (AGOWA, LGC Genomics, Berlin, Germany) and CSF microbiota amplicon library construction was carried out using a one-step PCR amplification targeting the V4 region of the bacterial 16S rRNA gene as described (Whitlock et al., 2021).

Sequencing of the pooled libraries was carried out for 600 cycles on an Illumina MiSeq desktop sequencer using the Miseq Reagent Kit v3.

### Whole Genome Amplification and Shotgun Sequencing

Whole genome amplification of DNA purified from CSF samples and two mock community samples was carried out using the REPLI-g Mini Kit (Qiagen, CN: 150023) in accordance with the manufacturer’s recommendations. For each sample, including mock communities and no-template controls, a random-fragment library was constructed using the Nextera DNA Sample Preparation Kit (Illumina) with dual indexing and sequenced on the HiSeq 2500 platform to produce 96-bp paired-end reads.

### Taxonomic Assignment of 16S Sequences With DADA2

Sequencing data were analyzed using the denoising program DADA2 (Callahan et al., 2016) (version1.6.0) as described (Nelson et al., 2019), and aligned to the SILVA 16S reference database (v. 132) (Pruesse et al., 2007) to produce a 16S amplicon taxa table for downstream computational analysis.

### Taxonomic Assignment of Shotgun Sequences With Kraken

Shotgun sequence data were analyzed using the program Kraken (Wood and Salzberg, 2014) against a custom database that included full genomic sequence of human, bacteria, virus, and archaea obtained from GenBank.

### *De Novo* Assembly and Annotation

Non-human DNA reads were assembled and annotated with the Comprehensive Genome Analysis service on the PATRIC website (Wattam et al., 2017). This analysis included assembly using SPAdes (Bankevich et al., 2012), a standard pipeline for annotating sequences with open reading frames and hypothetical function, and prediction of antibiotic resistance genes using the comprehensive antibiotic resistance database (CARD) (McArthur et al., 2013).

Multilocus sequence typing (MLST) analysis was performed on the PubMLST.org website (Jolley et al., 2018) for *S. epidermidis* (Thomas et al., 2007) and on Institute Pasteur MLST website (http://bigsdb.pasteur.fr) for *K. pneumoniae* (Diancourt et al., 2005).

The PATRIC Similar Genome Finder was searched with the assembled *S. epidermidis* CLIMB1 genome, and the closest 100 matching genomes were identified. A rooted phylogenetic tree was built using the Codon Tree method, with *Staphylococcus caprae* C87 as an outgroup.

## Results

### Sequencing-Defined Taxonomic Composition of CSF DNA

**Figure 1:**
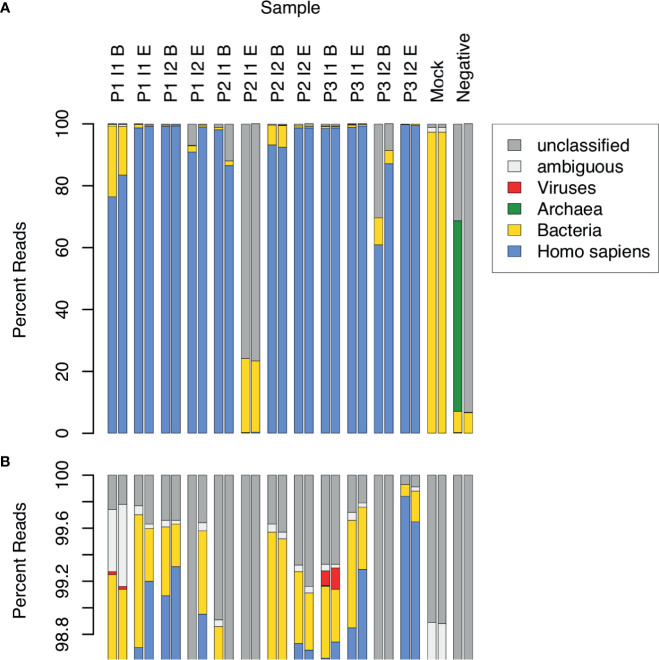
Relative abundance of sequence reads in CSF samples across major domains of life. 12 CSF samples were analyzed in duplicate by WGA. The samples came from 3 patients (P1, P2, P3), each having 2 infection episodes (I1, I2), with one sample collected near the beginning (B) and the other near the end (E) of the episode. Control samples consisted of a mock bacterial community (Mock) and a negative, no-template sample (Negative). Assignment of sequence reads to major domains of life was performed with Kraken: human (blue), bacteria (yellow), archaea (green), viruses (red), ambiguous, i.e. assignment to more than 1 domain (white), and unclassified, i.e. not assigned to any domain (grey). The lower panel **(B)** is an expanded view of **(A)**, showing the top percentile range and revealing 2 samples in which viral sequences were detected.

CSF samples were expected to contain substantial amounts of human DNA, along with DNA from other types of organisms, both pathogenic and opportunistic. We estimated the relative DNA abundance of organisms from the major domains of life in CSF samples by the distribution of reads in WGA (**Figure 1**). Human DNA constituted the overwhelming majority of reads in 10 out of 12 samples (range: 76.5 to 99.9%, mean: 95.4%, median: 98.7%). Two replicates of one sample (P3 I2 B) yielded 60.8% and 87.2% human reads, respectively, and another (P2 I1 E) yielded fewer than 0.5% human reads in both replicates. The low abundance of human DNA in these latter 2 samples, together with the detection of typical bacterial false positives (*e.g. Delftia* spp.*, Bradyrhizobium* spp.), indicated potential quality issues with the latter 2 samples. These samples were therefore excluded from further analysis. The proportion of sequencing reads contributed by bacteria in the remaining 10 samples ranged from 0.1 to 22.8% (mean: 3.1%, median: 0.6%). In 2 samples collected near the beginning of an infection episode, the proportion of bacterial reads was particularly high: Sample P1 I1 B – 15.7% and 22.8%, and P2 I2 B – 6.4% and 7.1%. Viral sequences were observed in 2 samples at low frequency (< 0.2%). No archaeal sequences were detected in any of the CSF samples.

**Figure 2:**
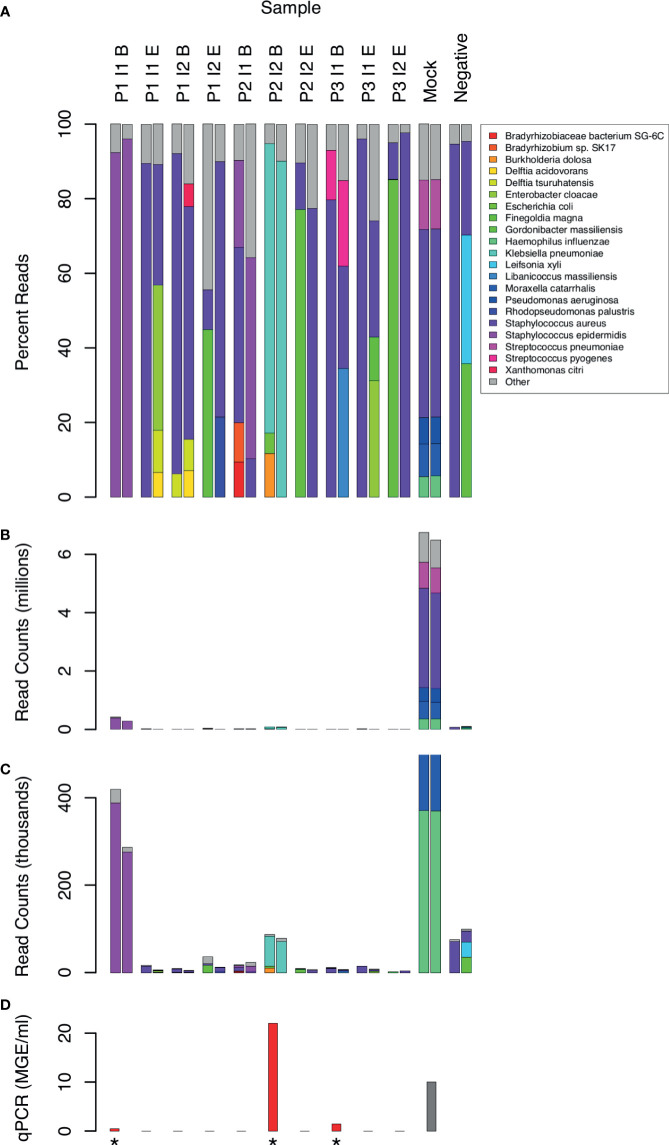
Distribution of bacterial species in sequence reads from 10 CSF samples. Sample labels are as in **Figure 1**. 2 samples with low human DNA content were excluded. 21 species that yielded 5% or more bacterial reads in at least one sample are shown by name. Less frequent species assignments are grouped into the “Other” category. Reads are plotted as percentages of bacterial reads **(A)** and counts **(B, C)**. Panel **C** shows the same data as **B**, but scaled to highlight the experimental samples, which had much lower counts than the mock positive control. qPCR measurements of 16S RNA are shown for comparison **(D)** in units of genome equivalents per ml. The gray bar (Mock) represents a computed, non-experimental value. Measurements above the limit of detection are marked with an asterisk.

Taxonomic assignment of WGA DNA reads identified 16 bacterial species that comprised 5% or more of bacterial reads in at least 1 CSF sample (**Figure 2**). Species with less than 5% of reads were assumed to be mostly artifactual and were excluded from the analysis. Although the Kraken software has excellent specificity (Lindgreen et al., 2016), it is difficult to distinguish true positives from contaminants. Consistency between replicates was limited even among species above the 5% threshold. Only the following species were found in both replicates of the same CSF sample: *S. epidermidis* in 2 samples (P1 I1 B and P2 I1 B), and *K. pneumoniae* (P2 I2 B) and *S. pyogenes* (P3 I1 B) in one sample each. *S. aureus* sequences were present in all samples, including the no template negative controls.

The absolute number of bacterial reads, although not a rigorous measure of abundance, was comparatively high in 2 samples (**Figure 2**, samples P1 I1 B and P2 I2 B). In both cases, the non-human reads were assigned to 1 predominant bacterial species (*Staphylococcus epidermis* in sample P1 I1 B and *Klebsiella pneumoniae* in P2 I2 B). These were also 2 of the 3 clinical samples yielding 16S rRNA above the level of detection by qPCR (**Figure 2**).

In addition to *S. epidermidis*, sample P1 I1 B had about 300 (0.02%) reads assigned to a *Staphylococcus* phage StB20-like genome. StB20 was previously described as a lysogenic bacteriophage isolated from a coagulase-negative *Staphylococcus capitis* (Deghorain et al., 2012). Sample P3 I1 B had 2000-3000 (0.1-0.2%) sequences mapped to multiple torque teno virus (TTV) genomes. TTV has been previously identified in CSF (Simner et al., 2018; Manso et al., 2020), but its clinical significance is unclear. Our preliminary findings from assembling the TTV reads indicate that the sample contained potentially 5 or more strains of TTV and torque teno mini virus.

### Comparison of Identified Microorganisms by Cultivation, 16S, and WGA

Identification of bacterial species by culture remains the gold standard for the microbiological characterization of CSF. Of the samples in this study, 6 were culture positive. We examined whether WGA and 16S data were consistent with culture results (**Table 1**). Culture made 7 bacterial species identifications in the 6 samples. Of these, WGA identified the same species as the most or second-most abundant in 5 cases. In the remaining 2 cases, the same species were present in the WGA data, but at an abundance too low to be called out as positives. 16S results resembled those from WGA, albeit at the genus and family levels. For example, *S. epidermidis* and *S. aureus* could not be distinguished by 16S.

**Table 1:** Comparison of taxonomic assignments of bacteria by microbiology lab culture, 16S, and WGA for 6 samples that were culture-positive.

Subject	Infection episode	Day of infection	Cultivation	16S			WGA		
			Taxonomic assignment	Taxonomic assignment	% Reads	Rank	Taxonomic assignment	% Reads	Rank
P1	I1	1	*Staphylococcus epidermidis*	*Staphylococcus*	98	1	*Staphylococcus epidermidis*	94	1
	I2	2	*Staphylococcus aureus*	*Delftia*	41	1	*Staphylococcus aureus*	78	1
				*Staphylococcus*	22	3			
P2	I1	1	*Staphylococcus epidermidis*	*Staphylococcus*	77	1	*Staphylococcus epidermidis*	41	1
	I2	1	*Klebsiella pneumoniae*	*Enetrobacteriaceae*	86	1	*Klebsiella pneumoniae*	83	1
			*Streptococcus mitis/oralis*	*Streptococcus*	0.01	22	*Streptococcus mitis*	0.002	110
P3	I1	2	Beta-hemolytic *Streptococcus species*, Group A	*Streptococcus*	90	1	*Staphylococcus aureus*	46	1
							*Streptococcus pyogenes*	13	2
	I2	1	*Staphylococcus epidermidis*	*Propionibacterium*	31	1	*Delftia acidovorans*	16	1
				*Staphylococcus*	5	8	*Staphylococcus aureus*	7	5
							*Staphylococcus epidermidis*	0.0004	446

For 16S and WGA the most abundant taxon is shown as well as the closest matching taxon to the other methods. Abundance is given as 2 measures, averaged over replicates: percent reads and rank when sorted by abundance.

### *De Novo* Genome Assembly

A set of contigs of sufficient quality was assembled *de novo* from one sample, P1 I1 B, yielding a draft *S. epidermidis* genome, which we refer to hereafter as CLIMB1 (see below). Assembly of the non-human reads from sample P2 I2 B yielded 55 contigs spanning 610 Kbp of DNA. 410 Kbp (68%) of this sequence showed over 99% identity to multiple *K. pneumoniae* chromosomal sequences from GenBank. 136 Kbp (22%) of the assembled sequence had a 100% match to an unnamed plasmid of 239 Kbp total length from *K. pneumoniae* KP14003. None of the 7 K*. pneumoniae* MLST genes were present in the assembled sequence. A possible explanation is that the contigs represent only a partial assembly of a putative *K. pneumoniae* genome, which missed the MLST genes by chance due to low coverage (< 10%) of the chromosomal sequence.

### Characterization of the *S. epidermidis* CLIMB1 Genome

**Figure 3:**
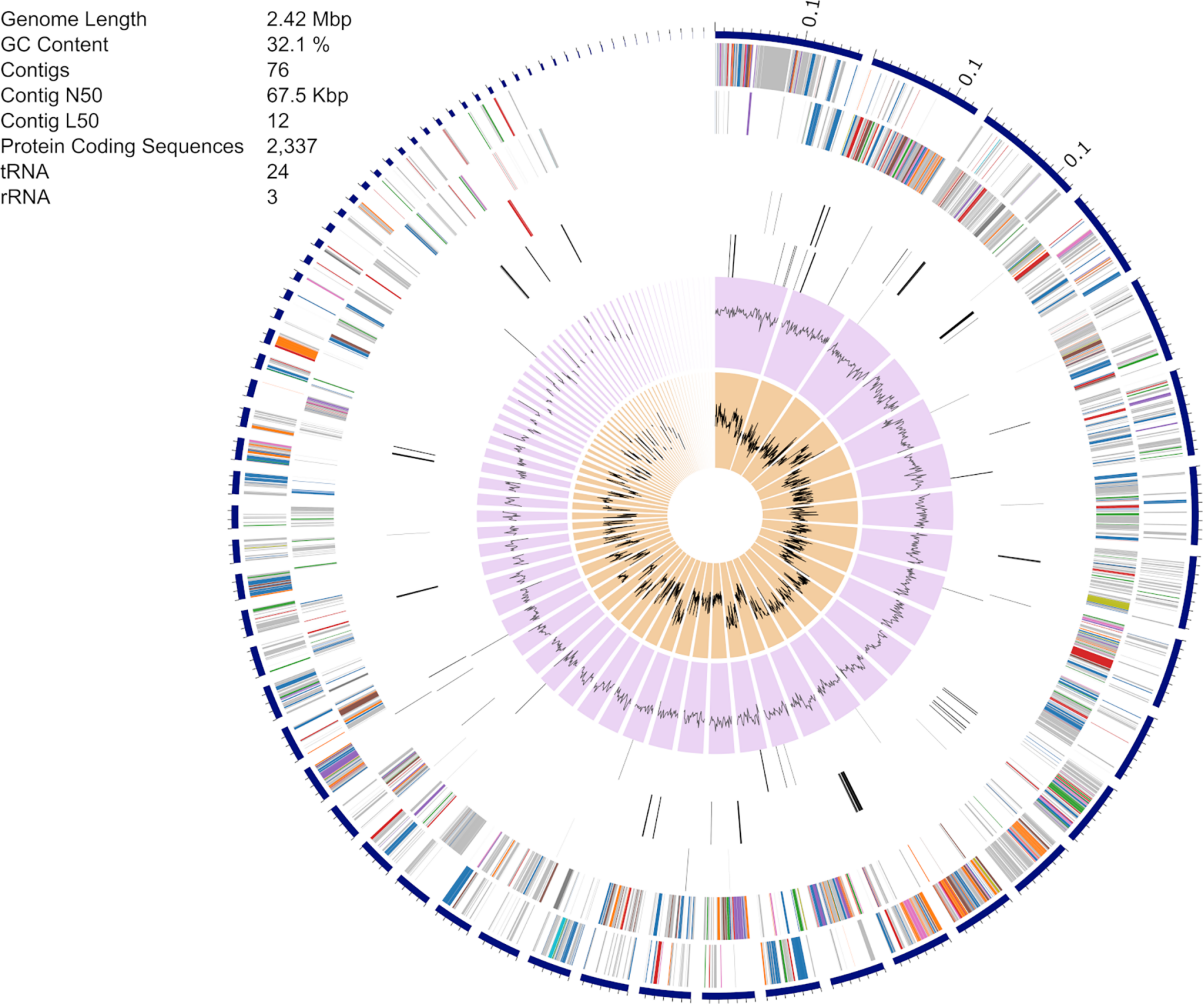
Assembly of the *S. epidermidis* CLIMB1 genome. The circular display of the assembly shows, from outer to inner rings, the contigs, coding sequences on the forward strand, coding sequences on the reverse strand, RNA genes, coding sequences of putative antimicrobial resistance genes, coding sequences of putative virulence genes, GC content, and GC skew. The embedded table includes summary statistics of the assembly.

The *S. epidermidis* CLIMB1 assembly consisted of 76 contigs comprising a total of 2.42 Mbp (**Figure 3**), in the range of typical *S. epidermidis* genomes (Conlan et al., 2012). The contig N50 value of 67.5 Kbp and L50 value of 12 reflects an acceptable quality of assembly. There were a total of 2,337 predicted protein coding sequences. Of these, 13 were annotated as putative antibiotic resistance genes based on a CARD database search (**Table 2**).

**Table 2:** Predicted antibiotic resistance genes in the *S. epidermidis* CLIMB1 genome ascertained from CARD.

Gene Name [Organism]	Drug Class
NorA [*Staphylococcus epidermidis*]	acridine dye; fluoroquinolone antibiotic
two-component response regulator [*Staphylococcus aureus* str. Newman]	acridine dye; fluoroquinolone antibiotic
aminoglycoside 3’-phosphotransferase (plasmid) [*Campylobacter coli* CVM N29710]	aminoglycoside antibiotic
streptomycin aminoglycoside 6-adenyltransferase, partial [*Streptococcus oralis*]	aminoglycoside antibiotic
dihydrofolate reductase [*Staphylococcus epidermidis* ATCC 12228]	diaminopyrimidine antibiotic
multidrug efflux pump (plasmid) [*Staphylococcus aureus*]	fluoroquinolone antibiotic
DNA gyrase subunit A [*Staphylococcus aureus* MRSA252]	fluoroquinolone antibiotic; nybomycin
macrolide 2’-phosphotransferase [*Staphylococcus equorum*]	macrolide antibiotic
Beta-lactamase [*Staphylococcus aureus* JH9]	monobactam; cephalosporin; carbapenem; cephamycin; penam
MecA [*Staphylococcus aureus*]	monobactam; cephalosporin; carbapenem; cephamycin; penam
streptothricine-acetyl-transferase [*Campylobacter coli*]	nucleoside antibiotic
beta-lactamase (plasmid) [*Staphylococcus aureus* USA300_TCH959]	penam
MarR family transcriptional regulator [*Staphylococcus aureus* ED98]	tetracycline antibiotic; peptide antibiotic; fluoroquinolone antibiotic; penam; cephalosporin; acridine dye

The *S. epidermidis* CLIMB1 genome assembly contained all seven MLST genes of *S. epidermidis* (*arcC*, *aroE*, *gtr*, *mutS*, *pyrR*, *tpiA*, *yqiL*). MLST sequences of CLIMB1 assign this strain to profile 16-2-1-2-2-15-1-1 or ST-16. The PubMLST database contains 3 other previously described isolates with profile ST-16, 2 from human(s) in the USA in 2001 and Russia in 2010 and 1 from an environmental source in Poland in 2007.

**Figure 4:**
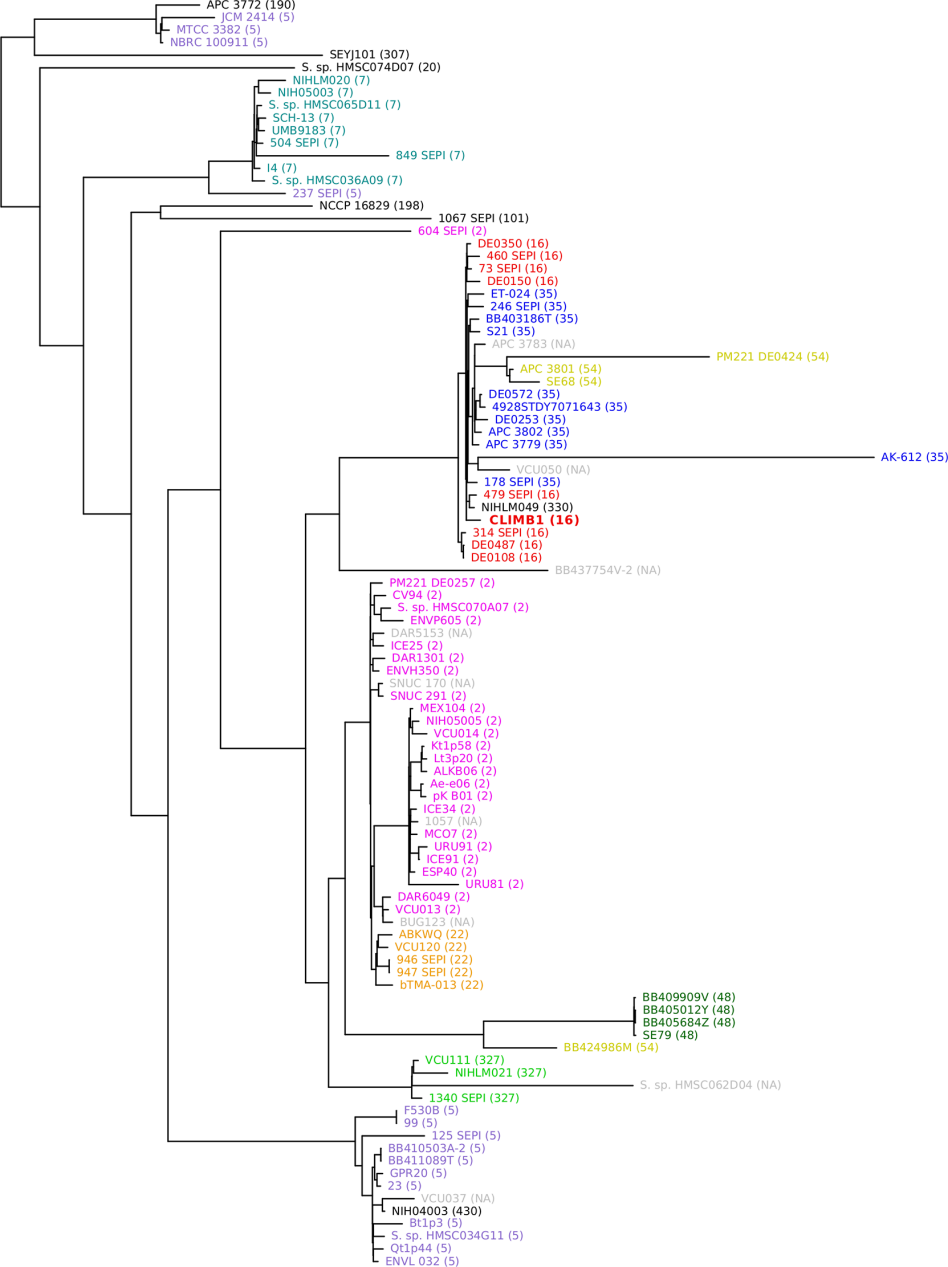
Phylogenetic tree of *S. epidermidis*, including CLIMB1 (red, center) and the most similar strains with known genomes, based on genomic sequence comparison. MLST profiles are indicated in parentheses and by color coding.

A phylogenetic tree was constructed with the *S. epidermidis* CLIMB1 genome and the 100 most similar genomes from the PATRIC database (**Figure 4**). Most of the included genomes were annotated as *S. epidermidis* strains. 6 genomes were described only as *Staphylococcus* strains. CLIMB1 belonged to a compact clade of 26 *S. epidermidis* genomes, 9 of which represented all strains with the ST-16 MLST profile. The clade included a variety of clinical and environmental isolates. One strain, NIHLM049, was described as a commensal strain that clustered with nosocomial isolates in a pan-genome study of 28 genomes (Conlan et al., 2012). A group of 7 genomes (DE prefix) were from environmental samples collected from the Duke University campus (NCBI BioProject PRJNA543692). Another 6 genomes (SEPI) were ICU isolates from neonatal or critical care units at the University of Washington Medical Center (Roach et al., 2015). 4 genomes (APC) were from isolates from human milk obtained in Ireland (Angelopoulou et al., 2020).

## Discussion

This proof of concept study showed the promise of WGA when applied to CSF shunt infection samples. WGA results were consistent with conventional microbiological culture and 16S. Dominant bacterial species observed in this study have been previously reported in CSF shunt infections (Simon et al., 2014a; Simon et al., 2014b; Whitlock et al., 2021). *S. epidermidis* and *S. aureus* are common occurrences, hypothesized to originate from the normal skin flora. *K. pneumoniae* was previously found in 1 CSF shunt infection patient (Simon et al., 2014b).

Compared to 16S, WGA provided resolution at the species or strain level for both bacteria and viruses. Compared to bacterial culture, both 16S and WGA identified additional organisms of potential clinical relevance. In some cases, the species recovered by bacterial culture were identified in 16S and WGA at only low relative levels. WGA data allowed us to assemble the entire genomic sequence of an *S. epidermidis* strain from a CSF shunt sample. Finally, WGA identified the presence of TTV viral genomes in CSF shunt infection samples, which neither bacterial culture nor 16S could detect.

WGA holds the promise of detailed molecular characterization of genomic DNA, which implies precise strain identification, evaluation of putative antibiotic resistance genes, and characterization of metabolic capacity. For example, the *S. epidermidis* genome we assembled with WGA/SS data yielded information about molecular strain type (MLST), antimicrobial resistance gene content, and phylogenetic relationships to previously identified strains. These results were achieved without physical removal of the highly abundant human DNA from CSF shunt infection specimens before processing for sequencing. However, this analysis was only possible for a sample with one predominant bacterial species, which yielded a sufficient number of sequencing reads. Therefore, the utility of WGA may be optimal at the beginning of CSF shunt infection, before the bacterial infection is cleared by antibiotic treatment.

This work builds on an earlier report of the application of WGA to a cohort of hospitalized patients with infectious meningitis and encephalitis (Wilson et al., 2019). In contrast to the earlier study, in which a minority (13%) of participants had hardware present, all children in the current cohort had CSF shunts. In addition, our study cohort had CSF shunt infection documented by bacterial culture, while WGA both confirmed culture diagnosis and identified potential additional bacterial and/or viral organisms present.

Because reinfection occurs at high rates among patients with CSF shunt infection, detailed genomic characterization of microorganisms in CSF can help answer questions about the etiology of infection and reinfection. We previously explored the relationship between infection and reinfection using 16S (Whitlock et al., 2021). In that study we showed that 16S results were generally consistent with culture-based methods and that 16S may detect organisms missed by culture at the end of infection treatment but detected by culture at reinfection. However, the CSF microbiota identified by 16S only weakly correlated within patients at the end of infection and beginning of reinfection. For this study, we explored whether WGA might provide critical information about the microbiota present at the end of infection. Instead, our results indicate that this method only yields useful results when sufficient microbial DNA is present, which occurs only at the beginning rather than the end of infection.

We therefore suggest a more targeted approach to address the question of microorganism persistence from one infection to another, which relies on the high resolution information provided by WGA and the enhanced sensitivity and specificity of PCR. The approach would apply WGA to CSF samples at the beginning of a reinfection to obtain the genomic sequence of the principal infecting organism. The sequence information would be used to develop strain-specific PCR primers, which would be used to detect the strain in late samples of the previous infection. A positive result would provide strong evidence for the hypothesis that microorganisms present at low levels in CSF during treatment of an infection can lead to reinfection. Such an approach would be based on existing methodology. For example, methods for designing specific primers based on bacterial genomic sequence have been described in the literature (Kechin et al., 2020). Nested PCR is a common technique for increasing sensitivity and specificity of DNA sequence detection (Green and Sambrook, 2019).

We acknowledge several limitations for this proof of concept study. Extraction and sequencing of DNA from CSF samples is challenging due to low DNA abundance. In addition, analysis of microbial sequences is limited by the high proportion of human DNA, even during an active infection. As a result, the sensitivity of WGA is not better than that of 16S and worse than culture. Only a small fraction of the CSF samples we analyzed produced a sufficient number of bacterial reads to allow partial genomic assembly of predominant species. An expanded study would be needed to better understand the applicability of the approach. WGA may only be useful for CSF infections where one or a small number of microorganisms dominate. Alternately, as has been shown in aseptic meningitis (Wilson et al., 2019), WGA may indicate the presence of other microorganisms, such as viruses present in the setting of CSF shunt infection. An additional consideration when choosing WGA is cost. Processing by 16S is estimated at $80/sample, while WGA can cost twice as much or more, depending on the depth of sequencing. Other factors that contribute to an increased cost of WGA are a potentially longer turn-around time for sequencing (3-6 days compared to 3 days for 16S) and a more complex computational pipeline.

Despite these limitations, our findings demonstrate the promise of WGA for research of CSF shunt infection. WGA results: (1) were consistent with culture-based methods, (2) identified all bacteria detected in culture to the species level, while taxa were classified by 16S rRNA to only genus or family level, and (3) provided additional insights regarding viruses present and strain identity of predominant bacteria. Further work is needed to better understand the utility of WGA in the setting of CSF shunts and CSF shunt infection, including a comparison between the effectiveness of the WGA with other approaches to characterizing the microbiota such as strain-specific PCR primers.

## Data Availability Statement

DNA sequence data presented in this study can be found online at the National Center for Biotechnology Information (https://www.ncbi.nlm.nih.gov) under BioProject PRJNA707514.

## Ethics Statement

The study received initial Institutional Review Board approvals from the Seattle Children’s Research Institute on Febryary 8, 2011 and the University of Utah, as well as approval from the Primary Children’s Hospital Privacy Board on January 14, 2009. For all study subjects, except those from Primary Children’s Hospital prior to March 18, 2010, consent was obtained for additional CSF to be collected on each occasion that regular CSF samples were obtained during treatment for CSF shunt infection. Prior to March 18, 2010 at Primary Children’s Hospital, we used CSF remaining for after routine processing and testing in the Primary Children’s Hospital Microbiology Laboratory.

## CLIMB Group Members

Current membership of the CLIMB group includes: Alexander Cheong, Gabriel Haller, Julie McGalliard, Diego Morales, Amanda Morgan, Alexander Rangel-Humphrey, and Lisa Wick. Past membership includes: Dan Berger, Whitney Bond, Haley Botteron, Courtney Dethlefs, Jessica Foster, Mohammed Gabir, Robert Johnson, Deanna Mercer, and Linda Shih.

## Author Contributions

TS, PH, CP, KW, and LH conceived and designed the study. DL, PM, JH, JO, and TS provided oversight over study enrollment and material collection. CP performed the experiments. PH, CP, and KW analyzed the data. PH, TS, and CP wrote sections of the manuscript. All authors contributed to the article and approved the submitted version.

## Funding

This study was supported by R01 NS095979. DL receives research funding and equipment for unrelated projects through Microbot Medical, Inc. and Medtronic, Inc. LH receives additional support from the National Institutes of Health *via* K24 HL141669.

## Conflict of Interest

Author KW was employed by company New Harmony Statistical Consulting LLC.

The remaining authors declare that the research was conducted in the absence of any commercial or financial relationships that could be construed as a potential conflict of interest.

## Publisher’s Note

All claims expressed in this article are solely those of the authors and do not necessarily represent those of their affiliated organizations, or those of the publisher, the editors and the reviewers. Any product that may be evaluated in this article, or claim that may be made by its manufacturer, is not guaranteed or endorsed by the publisher.
