# Supplementary figures and images for: Molecular Characterization of Microbiota in Cerebrospinal Fluid From Patients With CSF Shunt Infections Using Whole Genome Amplification Followed by Shotgun Sequencing
Source: Front Cell Infect Microbiol. 2021 Aug 20;11:699506. doi: 10.3389/fcimb.2021.699506 (PMC8417900; doi:10.3389/fcimb.2021.699506)

**Figure S1:** Procedural Flow diagram of 16S and WGA

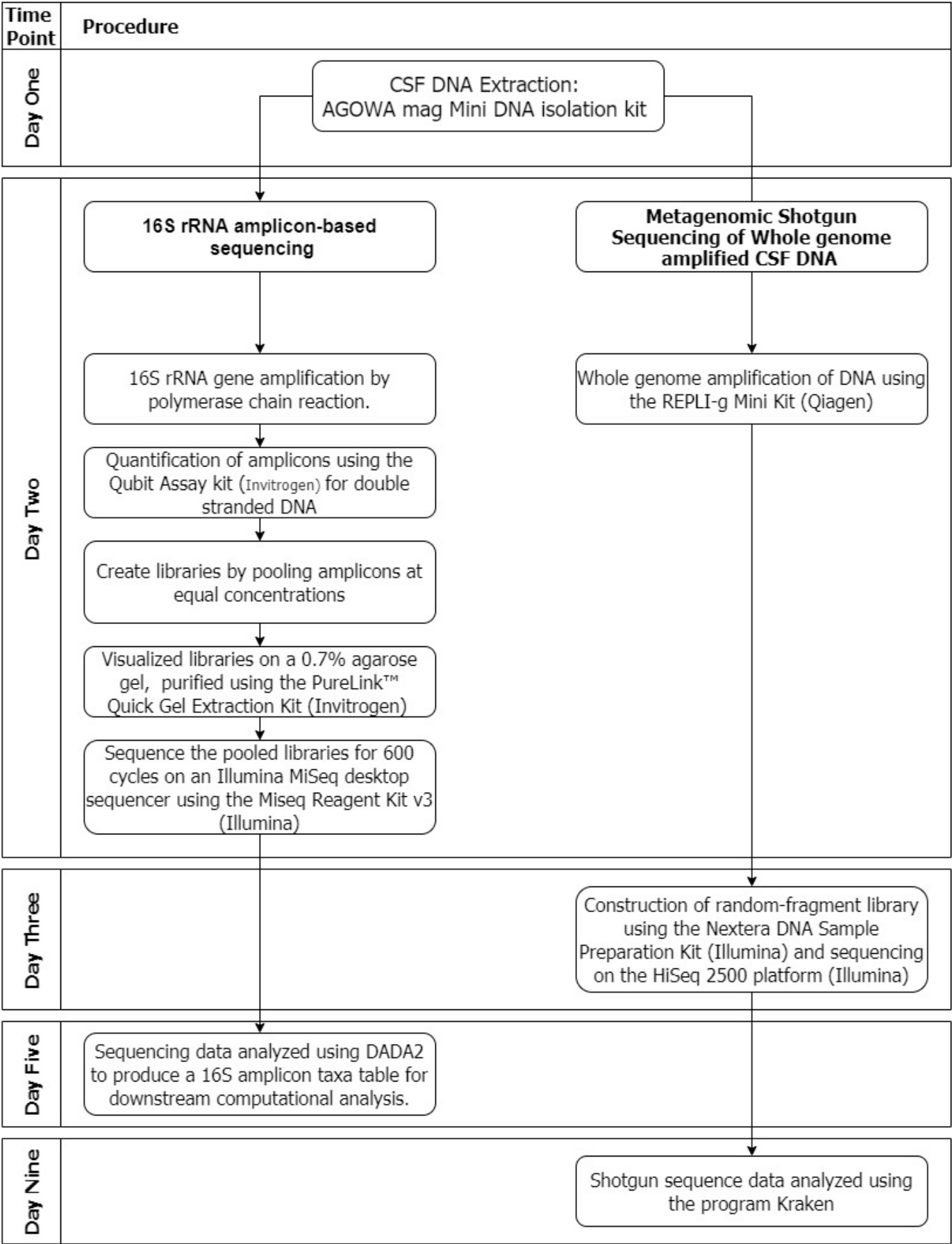

Supplement: Supplementary file 1 [file Image_1.pdf]
